# Value Coefficient of Polyethylene Fiber Soil Embankment Slope Based on Response Surface Analysis

**DOI:** 10.3390/polym14204295

**Published:** 2022-10-13

**Authors:** Yafeng Gong, Jiaxiang Song, Yulong He, Guirong Ma

**Affiliations:** 1 College of Transportation, Jilin University, Changchun 130025, China; 2 College of Civil Engineering, Hunan University, Changsha 410082, China

**Keywords:** polyethylene, fiber soil, embankment slope, response surface

## Abstract

The utilization of polymers can strengthen soil, but at a high price. In this study, value coefficients were proposed to evaluate the cost-effectiveness of fiber-reinforced roadbeds, and the effects of embankment-slope-influencing factors on the value coefficients were analyzed by response surface methodology. Ultrahigh-molecular-weight polyethylene fiber (UPEF) was used as the reinforcement material for soil. First, the shear strength parameters of fiber soil with different fiber diameters were obtained from the direct shear tests to set the parameters of the finite element models. Second, three factors, namely filling height, slope angle, and fiber diameter, were selected as input parameters based on the Box–Behnken Design (BBD) experimental design method, and their effects on the value coefficient of the fiber soil embankment slope were investigated. Finally, the design parameters at the maximum value coefficient of the fiber soil embankment slope were determined based on the results of the response surface analysis. The results indicated that the addition of UPEF could effectively improve the cohesion of the soil; the interaction between the filling height and fiber diameter is most obvious. The optimization of design parameters based on the value coefficient of the fiber soil slope is a slope-engineering design method considering comprehensive benefits.

## 1. Introduction

High-grade roads require a high load-bearing capacity for embankment slopes, and nearby available soils may not always meet the requirements of high-grade roadbed soils. Therefore, soil often has to be reinforced to meet the relevant design criteria. Reinforcement methods for embankment slope soils are mainly divided into chemical stabilization and physical reinforcement [1]. Chemical stabilization is achieved by adding additives, such as cement and lime, to soil to improve its strength and stability [2]. However, there are some disadvantages to chemical stability. For example, the pH of the soil changes after the additives are mixed with the soil, and this will cause environmental pollution [3]. Therefore, methods of physical reinforcement are increasingly being chosen. Research related to fiber-reinforced soils as a physical-reinforcement method has been carried out extensively [4].

Fibers for soil reinforcement include natural fibers and synthetic fibers. Recently, many studies have been conducted on the use of natural fibers as soil reinforcement materials to improve the physical and mechanical properties of soils due to their advantages such as wide distribution of resources, good economic benefits, and environmental protection [5,6]. The incorporation of coir fiber into soil not only enhances its strength and stiffness [7] but also reduces the seepage velocity of the soil, thereby increasing the piping resistance of the soil [8]. Fine-grained soil mixed with jute fibers can improve its unconfined compressive strength under freeze–thaw cycles [9]. In addition, jute fibers with a content of 0.6% and a length of 6 mm have been added to expansive soil to effectively improve its shear strength [10]. Palm-fiber-reinforced silty sand with a fiber length of 30 mm and fiber content of 0.5% has the best shear-strength reinforcement performance [11]. The shear strength and deformation of silty clay can be improved with the addition of sisal fibers [12]. The addition of corn silk and corn starch to soft soils can improve their compaction properties, and the optimal contents of the additive are 0.5% corn silk and 4% corn starch [13]. A series of free pressure tests was conducted to examine the effects of human hair fibers on the reinforcement of clay soils under freeze–thaw cycles, and the results showed a significant increase in unconfined compressive strength due to the addition of 1.5% human hair fibers [14]. Similarly, when adding wool to clay, the fiber content of 1.5% remains the optimal content for compressive-strength enhancement after freeze–thaw action [15].

Despite their impressive performance, natural fibers generally have little moisture and few biodegradable characteristics, and this can affect the life of the fiber and increase the cost of the project [16]. Therefore, polymer fiber has gained momentum as one of the high potential reinforcement materials in road engineering due to its stable and easy construction [17]. Currently, polypropylene fibers and polyethylene fibers are commonly used in engineering. Diambra [18] conducted an experimental study on the addition of polypropylene fibers to Hostun RF sand with different densities and found that the triaxial compressive strength of fiber-reinforced sand increases significantly with the increase in polypropylene fiber admixture. A finite element model of the polypropylene fiber soil embankment slope was established, and the effect of freeze–thaw cycling on it was analyzed by Gong and He [19]. A series of consolidated drained triaxial tests were carried out by Med Bouteben to obtain the mechanical parameters of the polypropylene fiber-reinforced cement–sand soils, which were applied to the numerical analysis of the embankment finite element model [20]. Li conducted a model roadbed test to investigate the effect of polypropylene fiber reinforcement on the settlement of the model roadbed, and the experimental results illustrated that the settlement of the fiber-reinforced soil roadbed is substantially reduced under high pressure [21]. Akbulut used polyethylene fibers as a reinforcing material for clay and found that polyethylene fibers can effectively increase the strength of clay [22]. Notably, from the perspective of environmental protection, some scholars stirred waste bags or plastics with polyethylene as the main component into soil, and this method was found to increase the shear strength and deformation resistance of the soil to a certain extent [23,24]. However, the research results showed that the reinforcement effect of waste polyethylene fiber is poor, and the quality is difficult to be unified. Therefore, waste plastic as soil-reinforcement material is not recommended [25].

**Figure 1:**
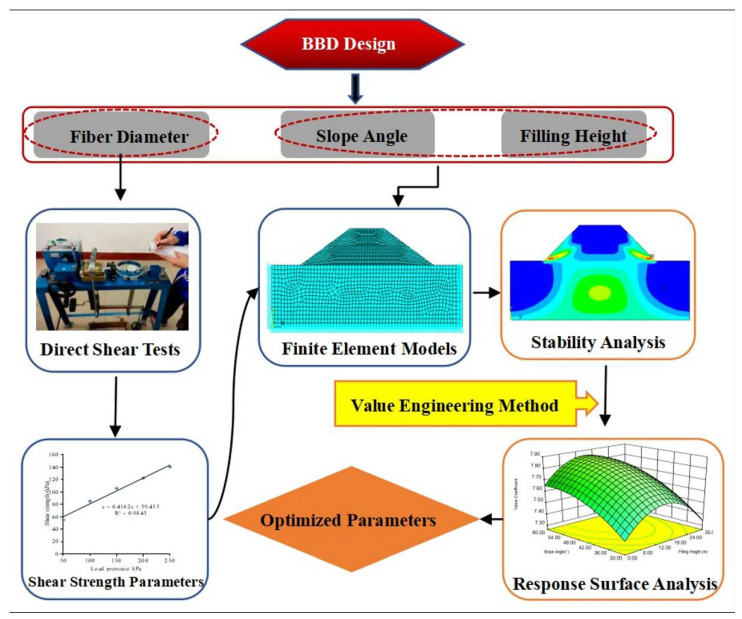
Technology roadmap.

In embankment-slope filling, the strength of the fiber-reinforced soil and the resistance of soil to deformation are critical [26]. The increase in strength can improve the stability of the slope, and the high modulus fiber can reduce the settlement of the slope. The optimal content is sought to achieve maximum strength. However, from the perspective of comprehensive benefits, higher fiber content means higher cost, which leads to poorer economic benefits, even though the structural load-bearing capacity is increased. Therefore, a comprehensive benefit evaluation method of fiber soil slope based on the value engineering method is proposed in this paper. The research process is shown in Figure 1. On the basis of the material parameters obtained from the direct shear test, a finite element model of the fiber soil embankment slope was established to analyze the stability of the slope model. From the perspective of cost-effectiveness, the response surface model was established based on the value coefficient of the fiber soil slope, and the best design parameters were fitted by the model.

## 2. Direct Shear Test

### 2.1. Materials and Sample Preparation

Soil sampling was performed in a subgrade fill in the northeast seasonal freezing area, China, which is yellowish in color and has a certain cohesiveness. Prior to the direct shear test, a series of tests were performed on the soil samples to obtain the basic physical properties of the soil, and this included specific gravity tests, compaction tests, and limit water-content tests. The basic physical properties of the soil samples are summarized in Table 1.

Ultrahigh-molecular-weight polyethylene fiber (UPEF) is a high-performance fiber with high specific strength, high modulus, and corrosion resistance, which can effectively improve the strength and deformation resistance of fiber soil as a reinforcing fiber. The UPEF used in the test was made by Shandong Fiber Building Materials Technology Co., Ltd, Qindao, China. It is slightly white and has good flexibility. The molecular weight of UPEF is 1.5 million. UPEF has a density of 0.98, very high tensile strength (3300 MPa) and elasticity modulus (95 GPa), and good high-temperature stability (melting point of 160 °C). The diameter of the fiber is an important factor for the shear strength, and the UPEF with different diameters can affect the project cost. The commonly used UPEF diameter specifications in the market are 0.02, 0.12, and 0.2 mm. In the direct shear test, the fiber content with the mixing ratio of 6‰ and the fiber length of 9 mm were selected based on previous research and pre-experiments [4]. Therefore, after precise cutting, the direct shear test was performed with the UPEF of 9 mm in length.

### 2.2. Test Procedure

The first step was to prepare the test specimen. The compaction test was conducted to determine the maximum dry density and the optimum water content of the test soil, which was the target water content of the direct shear test specimen. First, the initial soil was dried, crushed, and dried at 105 °C to completely evaporate the water. Second, the dried soil was sieved through a 0.5 mm sieve for the cylinder test piece of the direct shear test. The dry soil, target moisture content of water, and UPEF were mixed well in a cement mortar mixer made by Jinrui Test Instrument Co., Ltd, Cangzhou, China after 2 min of mixing. The variables for this test were fiber diameters of 0 (plain soil), 0.02, 0.12, and 0.2 mm. Compaction of 0.96 was achieved by using hydrostatic forming, according to the test protocol. Finally, the direct shear specimens were tightly wrapped with cling film to ensure constant moisture.

The prepared specimens were subjected to direct shear tests. The shear stress of the soil at the time of damage was obtained from the direct shear test, and the internal friction angle and cohesion of the soil were calculated based on the Mohr–Coulomb law. The shear strength of four main groups of soils was measured by the direct shear test. The four groups comprised plain soils and fiber-reinforced soils mixed with different UPEF diameters (0.02, 0.12, and 0.2 mm). In the direct shear test, each test group was composed of six test pieces with applied vertical loads of 50, 100, 150, 200, 250, and 300 kPa. During the test, when the horizontal displacement value started to decrease, the specimens were considered damaged, and the shear stress value at this time was recorded as the shear strength. The test procedure was performed according to the relevant code [27].

### 2.3. Test Results

Direct shear tests were performed in line with the test protocol, and the peak shear stresses obtained were processed. The shear strengths of the specimens with different pressure values were linearly fitted to obtain the shear strength parameters, *c* and *φ*, which were calculated by Equation (1):

(1) τ=σtanφ+c

The shear strength parameters of fibrous soils with different UPEF diameters are presented in Table 2.

A pattern was observed in the data in Table 2; the variation in fiber diameter had little effect on the internal friction angle of UPEF-reinforced soil, but it could effectively enhance cohesion. The main strengthening mechanism of UPEF was still the reduction of shear interface slip caused by the friction between soil particles and fibers [28]. Therefore, as the fiber diameter increased, the direct contact area between soil particles and fibers increased, and the increasing interfacial friction was the main factor for the increasing cohesion. The cohesion enhancement of the fiber soil was not effective as the fiber diameter exceeded 0.2 mm. Although the interfacial shear strength between a single fiber and the soil increased, the increase in fiber diameter caused a decrease in the number of interfacial shear fibers, because the fiber content was certain [4].

## 3. Finite Element Analysis

### 3.1. Computational Model

**Figure 2:**
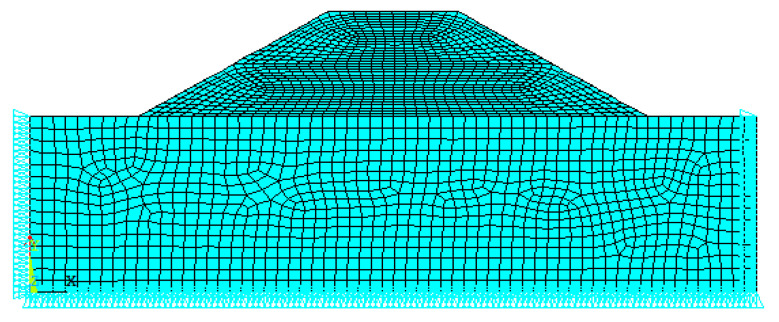
Finite element model of the embankment slope.

The numerical model of fiber soil embankment slope was established by the general finite element software ANSYS 12.1. The longitudinal length of the embankment slope is longer than the transverse width, and the disease of the embankment generally occurs mainly on the outward slope. Therefore, the embankment model can be simplified to plane model analysis [19]. In the calculation model, the height of the foundation was 50 m, the top width of the embankment was 36 m, and the fill height and slope angle were the geometric variables of this study. The geometric model was established in ANSYS and meshed; boundary constraints and loads were applied. The finite element model with a slope angle of 30° and an embankment filling height of 30 m is presented in Figure 2.

Before analyzing the finite element model of an engineering structure, the element type and material parameters of the element must be determined. Since the longitudinal length of the long slope is much larger than the transverse size, the slope stability problem can be classified as a typical plane strain problem. Plane 182 element is a two-dimensional element model with eight nodes; it is a favorable element to reflect the stress state and deformation of soil [19]. The element density was set to 1.725 g/cm^3^, which was the maximum dry density of the soil obtained from the compaction test. In addition, the constitutive model of soil was established. The Drucker–Prager (D–P) model was selected as the soil constitutive model of embankment slope, and it is a commonly used constitutive model to simulate slope soil in ANSYS software. There are two important soil shear strength parameters in the D–P model: cohesion (C) and internal friction angle (φ), which were derived from the direct shear test (Table 2).

The finite element geometric model was established based on the geometric parameters. For plane strain problems, Plane182 element has good adaptability. In mesh generation, each side of the model was divided into at least 10 equal parts to ensure sufficient accuracy based on the results of the pretest. By uniform division, the non-convergence of model calculation caused by triangular elements and sharp element angles can be avoided. After the mesh division of the model, the constraint conditions and loads were set. For both sides of the foundation boundary, the horizontal displacement was constrained, and the lower boundary was fully constrained. In addition to setting the self-weight of the embankment slope as the loading condition, a strip load of size 10.5 kN/m was applied on the top of the embankment to simulate the traffic load according to the design specification [29].

The stability of the embankment slope was analyzed by the reduction coefficient method. The shear strength parameters (cohesion and internal friction angle) of the tested slope soil were converted by the reduction coefficient. On the basis of the reduction coefficient method, the stability of the embankment slope was analyzed. The shear strength parameters (cohesion and internal friction angle) of the tested slope were converted by the reduction coefficient method. After selecting an initial reduction coefficient, *F*, the internal friction angle, *c*′, and cohesion, *φ*′, of the reduced slope soil mass were calculated according to Equations (2) and (3):

(2) $$c’=\frac{c}{F}$$

(3) $$tan\varphi^{\prime }=\frac{tan\varphi }{F}$$

where *c* and *φ* are the initial cohesion and internal friction angle of the soil, respectively.

For slope stability calculation, the discounted shear strength parameters were substituted into the finite element model for calculation. If the calculation results converged, the slope was stable. The reduction coefficient increased for numerical calculations until the results diverged, at which point, *F* is the stability coefficient of the slope [30].

### 3.2. Stability Analysis

After the analysis was completed, the results, such as strain and displacement, were viewed through the postprocessing module of ANSYS. The calculated results of the plain soil slope and the UPEF slope with 0.2 mm fiber diameter at a slope angle of 45° were selected for comparison to illustrate the contribution of adding fibers into the soil to the slope stability of the embankment.

**Figure 3:**
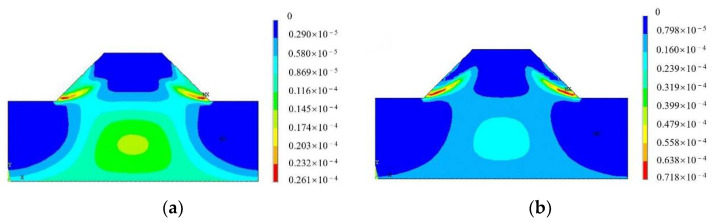
Plastic strain diagram with reduction coefficient of *F* = 1.4. (**a**) Plain soil slope. (**b**) Fiber soil slope.

After performing stability analysis and solution, viewing the results in ANSYS postprocessing revealed that the horizontal displacement in the X-direction changed as the plastic strain developed faster with the increase in the reduction coefficient. When the reduction coefficient of the plain soil slope increased to 1.5, the calculation result showed no convergence, which demonstrated that the slope was unstable. The stability coefficient of the plain soil slope was 1.4, but the stability coefficient of the UPEF slope was 1.7. The plastic strain of the embankment slope under different fills when the reduction coefficient, *F*, was 1.4 is shown in Figure 3.

As shown in Figure 3, the plastic strain of the plain soil slope developed more significantly compared with the fiber soil slope when the reduction coefficient, *F*, was 1.4. The plastic strain in the plain soil slope extended upward from the foot of the slope, and a narrow plastic strain zone was about to run through the whole slope, which would make the slope unstable. The increase in shear strength of UPEF soils resulted in better stability of embankment slopes, and this result was more significant in practical engineering, considering the local restraint effect of UPEF on the soil.

**Figure 4:**
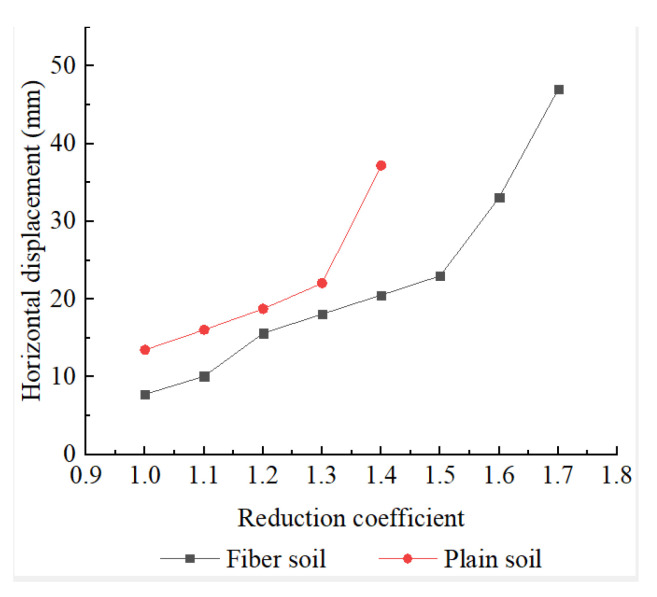
Extreme values of displacement in the x-direction of embankment slope with different reduction coefficients.

The x-directional displacement extremums for each strength reduction factor are shown in Figure 4. The x-directional displacement increased continuously as the reduction coefficient increased and the shear strength decreased. The x-directional displacement extremums of slope increased rapidly when it was close to instability, so there was a critical point to reflect the accelerated increase of the x-direction displacement of the slope at a certain reduction coefficient. The critical point was 1.3 for plain soil slopes and 1.5 for UPEF slopes, and the extreme value of x-direction displacement for UPEF slopes was always smaller than that for plain soil slopes with the same reduction coefficient, which fully demonstrated the high stability of UPEF slopes.

## 4. Response Surface Analysis

### 4.1. Theory and Method

#### 4.1.1. Response Surface Method

The response surface method is a parameter optimization method for experimental design and statistical analysis proposed by Box and Wilson [31] in 1951 that has good robustness [32]. The response surface method was used for data analysis, which combined the experimental design method with data analysis and statistics for optimization and used explicit polynomial expression to express the implicit function. In the response surface method, the relationship between the imported variable and the response value is explained by the following Equation (4):

(4) $$y=f\left(x_{1},x_{2}\cdots x_{i}\right)+\varepsilon$$

According to the Taylor formula, the response value, *y*, can be fitted by the response variables, *x*_1_, *x*_2_ … *x_i_*, with polynomial functions, and *ε* is residual. A common quadratic polynomial equation is represented in Equation (5) [33]:

(5) $$Y=\beta_{0}+\sum_{i=1}^{k}\beta_{i}x_{i}+\sum_{i=1}^{k}\beta_{ii}x_{ii}{}^{2}+\sum_{i=1}^{k-1}\sum_{j=i+1}^{k}\beta_{ij}x_{i}x_{j}+\varepsilon$$

where *Y* represents the shear strength parameters of soil, and β is the undetermined coefficient estimated by the polynomial fitting function; the most commonly used estimation method is the least square method; $\beta_{0}$ is a constant; $\beta_{i}$ (*i* = 1, 2, 3) is the linear coefficient; $\beta_{ii}$ is the quadratic coefficient of $x_{ii}$; and $\beta_{ij}$ is the interaction coefficient.

The accuracy of the response surface was determined by mathematical statistics. Analysis of variance (ANOVA) was used to assess the variance of the test sample data. Response surface models were fitted in accordance with the value coefficients calculated for different embankment slope models. ANOVA and interaction were used to determine the effect of different geometric parameters and fiber incorporation on the slope value coefficients.

#### 4.1.2. Value Engineering Method

The value engineering method is a commonly used method to obtain the best cost–performance ratio. By calculating the value coefficient of the research object, the most cost-effective conditions of use in the study were determined. For embankment slope engineering, the value engineering method mainly considers three aspects for parameter design: cost performance ratio (*V*), safety stability coefficient (*FI*), and project cost (*CI*). The relationship among these aspects is shown in Equation (6) [34].

(6) $$V_{i}=\frac{FI_{i}}{CI_{i}}$$

where *V* is the value coefficient, *FI* is the function coefficient, *CI* is the cost coefficient, and *i* is the label of the implementation scheme for completing the product.

There are specific requirements for the functional requirements of the project in different engineering environments, so the functional coefficient in value engineering should be specified in advance. In the analysis of the slope, the function coefficient is mainly determined by considering the safety and stability of the slope. The cost coefficient of this study mainly considered the cost of materials used to fill the slope. In this paper, the main influencing factor of the project cost was the amount of fiber. The total cost was obtained by multiplying the cost of fiber per unit area and the total area. The value coefficients were selected to judge the cost effectiveness of different slope-filling solutions, as well as to determine the most cost-effective parameter design solutions based on the value coefficients of slopes with different design parameter conditions.

### 4.2. Response Surface Test Result

The Box–Behnken Design (BBD) is the most widely and commonly used design in response surface methodology due to its fewer experimental requirements and excellent results [35]. The stability coefficient of the fiber soil embankment slope was analyzed by the value engineering calculation method. A higher value coefficient indicates that it has a relatively high safety coefficient at a lower price cost, which conforms to the principle of safety and economy in engineering design. In this paper, the slope angle, filling height, and fiber diameter were used as the response variables, and the value coefficient was used as the response value to evaluate the comprehensive economy of the UPEF soil embankment slope under the corresponding design parameters. The corresponding response surface fitting equation was established based on the calculation results obtained by the value engineering method.

It is worth noting that the commonly used design parameters are mainly considered as analysis variables when selecting parameters. In this paper, there are three design parameters, namely fill height, slope, and fiber diameter. These three factors affect the geometry and material parameters of the finite element model and are related to the slope stability and cost. The fill height and slope angle were commonly used design values for slopes, and the design value of fiber diameter was also the most commonly used value in the market. Three levels of the three design parameters were set. The slope angle of the embankment slope was designed between 0.3 and 0.6; the fiber diameters of 0.02, 0.12, and 0.2 mm were used. The fiber filling height was 0-layer filling, half filling, and all filling in three ways. The response surface test scheme was designed by using the professional test design software Design Expert. A response surface design with three levels and three factors was obtained by using the BBD experimental design method. The design of the response surface test protocol requires the coding of the influencing factors. The high, medium, and low values of the influencing factors correspond to the codes 1, 0, and −1, respectively, and the coding table is shown in Table 3. The response surface test protocols designed based on Table 3 are listed in Table 4, which also contains the response values (value coefficient) corresponding to the different run numbers.

### 4.3. Statistical Analysis

The value coefficients obtained from the analysis were fitted, and a trivariate regression fitting formula was obtained as shown in Equation (7):

(7) $$\begin{array}{l} F=7.83-0.091\times A+0.022\times B-11.93\times C-2.28\times 10^{-5}\times AB+1.1\times AC\\ -0.027\times A^{2}C+0.21\times BC-1.97\times 10^{-3}\times A^{2}-3.39\times 10^{-4}\times B^{2}-10.19C^{2} \end{array}$$

The accuracy of the value coefficient response surface of fiber soil slope was determined by statistical method. ANOVA is a commonly used method to evaluate the difference in value coefficient changes caused by design parameters. A fitted regression model expressed by Equation (9) was constructed based on the value coefficient calculation results provided in Table 4, and the accuracy of the model simulation was determined by ANOVA. The value coefficients in Table 4 were entered into Design Expert for ANOVA, and the ANOVA results are presented in Table 5.

The F-test was performed on the regression fit equation, and the magnitude of the discriminated *p*-value determined the significance of the fitted equation; the smaller the *p*-value, the higher the significance [36]. The Model F-value of 18.37 implied that the model was significant. There was only a 0.10% chance that a “Model F-value” this large could occur due to noise. Values of “Prob > F” greater than 0.10 indicated that the model terms were not significant. Values of “Prob > F” less than 0.05 indicated that model terms were significant. In this case, A, C, AC, BC, A^2^, and A^2^C were significant model terms.

The above ANOVA on the response surface model of value coefficient showed that the slope angle, filling height, and parameters of fill material had a large influence on the value coefficient of the fiber soil slope. The analysis of the F-value demonstrated that the fiber diameter had the greatest significance on the value coefficient, and the UPEF soil filling height had the lowest F-value; therefore, its influence was relatively less significant. The F-value of the multiple-times term was larger, thereby indicating that the influence of several influencing factors on the value coefficient had a non-linear influence.

**Figure 5:**
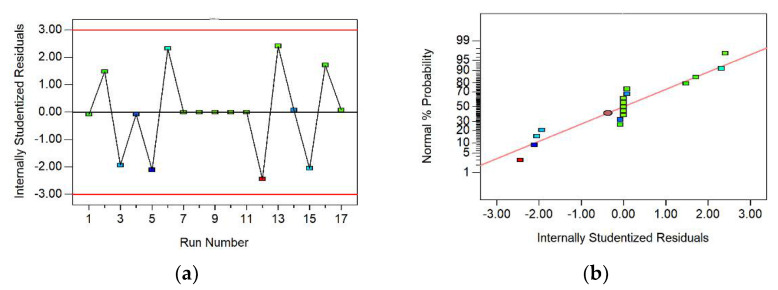
Residual distribution diagram of the response variable. (**a**) Internally studentized residuals vs. run number (The red line is the residual range line). (**b**) Normal probability vs. internally studentized residuals (The red line is the residual regression line).

**Figure 6:**
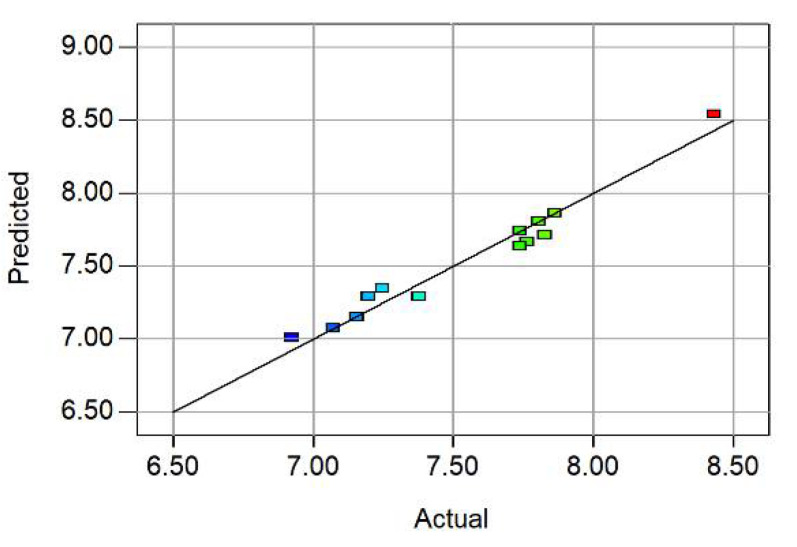
Comparison of calculated and predicted values (The black line is a 45 degree diagonal).

In the analysis process of the response surface method, the accuracy of the regression equation was verified by testing the residual normality distribution of the response values (value coefficients) and by comparing the predicted values with the analyzed values. These inspections are shown in Figure 5 and Figure 6.

The residual value level is also a powerful condition to reflect the quality of the mathematical model. The closer the residual normal distribution plot is to a straight line, the denser the observations near the regression line are, and the better the fit is [35]. A probability plot of the normal distribution of residuals for the response surface model was plotted (Figure 5). The value coefficients of the fibrous soil slopes were mainly distributed on a sloping straight line, which indicated that the results of the value coefficient analysis showed an approximately normal distribution. In addition, the residual distribution indicated that the residuals were usually random and showed the accuracy of the model [37].

Figure 6 shows a good fit for the response surface model. According to Figure 6, most of the analyzed data points were concentrated above the 45-degree sloping straight line, and only a few calculated points were discrete, which showed that the actual values were very close to the predicted values of the model. Therefore, the fitted equation obtained by regression, using the response surface method, could accurately predict the value coefficient of the fiber soil slope.

In addition, the applicability and significance of the model were checked by various statistical factors in Table 6. The coefficient of variation (CV) is the ratio of the standard deviation (SD) to the mean, which is a normalized measure to reflect the degree of dispersion of the model. In this case, the coefficient of variation was 1.49%, which indicated the high accuracy of the model. R^2^ is a common metric used to test predictive models, comparing how well the predicted results match the actual occurrence. The proposed model showed an R^2^ of 0.9684, which was a high value indicating a strong agreement between the predicted and true values based on the response surface. “Pred R-Squared” was a negative value, which implied that the overall mean was a better predictor of response than the current model.

**Figure 7:**
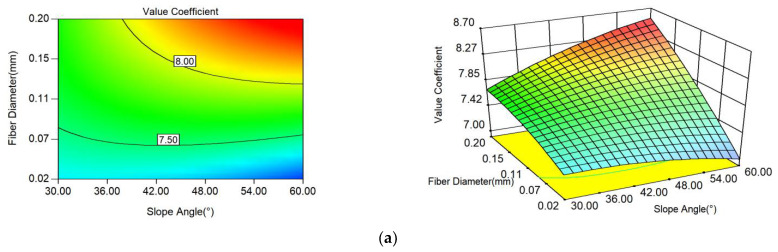

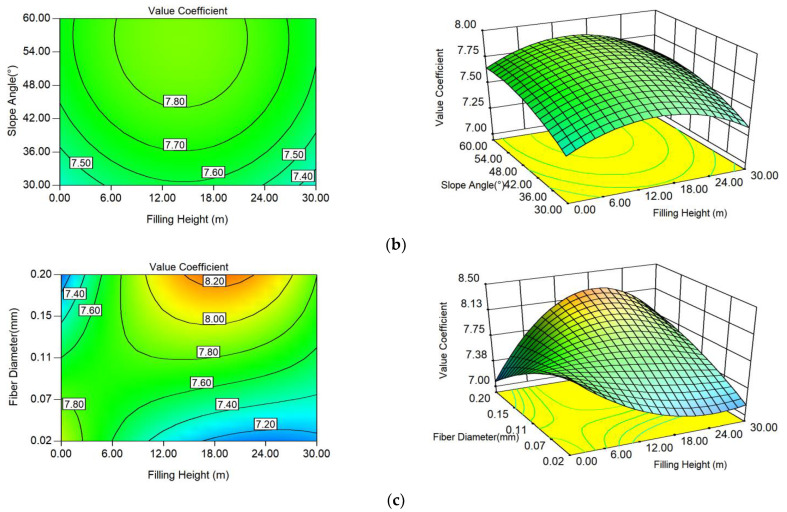
Response surface and contour plots. (**a**) Interaction between fiber diameter and slope angle. (**b**) Interaction between filling height and slope angle. (**c**) Interaction between filling height and fiber diameter.

The value coefficient was not only related to the safety coefficient but also closely related to the project cost. The three factors were closely related to the above two. The interaction between factors was studied to determine the change in response value under the coupling of multiple factors. The response surface and contour map could intuitively reflect the impact of interaction on the response value. The steeper the surface and the denser the contour, the more significant the impact, and the stronger the interaction between the two factors. The contours of the factor interactions and the response surfaces are shown in Figure 7.

Figure 7 shows that the three influencing factors of slope angle, filling height, and incorporated fiber diameter had a significant interaction; the interaction between the filling height and fiber diameter was the most obvious. As the fiber diameter increased, the safety coefficient of the soil increased, but so did the cost; the increase in filling height not only changed the value coefficient caused by itself, but it also made the interaction more pronounced due to the fact that the increase in filling height was accompanied with an increase in fibers. The interaction between the fiber diameter and slope angle was very strong, whereas the interaction between the fill height and slope angle was strong to some extent. Thus, each influencing factor did not independently affect the value coefficient of UPEF soil slope, and this result was consistent with the ANOVA results of the interaction term.

In the interaction between fiber diameter and slope angle, the response surface was mainly an oblique upward surface, because the value coefficients all increased with the increase in the influencing factor. Its oblique direction was mainly toward the direction of fiber diameter, so the influence of fiber diameter on the value coefficient was large. The response surface of filling height versus slope angle was spherical, and the contour arrangement was sparser when the slope angle of the side slope was larger. In the interaction between filling height and fiber diameter, the response surface was a complex surface similar to the saddle surface, the contour arrangement was very dense, and the interaction between the two was very significant. The value coefficient increased and then decreased as the filling height increased, and this indicated that the interaction of the two factors had a complex effect on the value coefficient.

The above contour map and response surface map showed that the contour map under the interaction of the three influencing factors in the whole boundary range was in the shape of opening, thus showing that the extreme value of the UPEF slope value coefficient may not appear in the selected parameter design range. On the basis of the obtained response surface equation, combined with the optional conditions for the design value coefficient of fiber soil slope, the optimal design parameters were obtained. The optional conditions were set, as shown in Equation (8):

(8) $$\begin{array}{cc} \mathrm{Max}\mathrm{FT}= & f\;(A,\;B,\;C)\\ & s.t.\\ & 30\geq A\geq 0\\ & 60\geq B\geq 30\\ & 0.2\geq C\geq \mathrm{0.02} \end{array}$$

In the above equation, FT is the fitted response value; A, B, and C are the filling height, slope angle, and fiber diameter, respectively. As shown in Equations (9) and (10), the optimal design parameters for determining the soil slope based on the value coefficient of UPEF soil slope were obtained as follows: the filling height of 18.52 m, the fiber slope angle of 56.11°, and the fiber diameter of 0.19 mm (value coefficient of 8.48).

## 5. Conclusions

The shear strength parameters of UPEF soil were measured through direct shear tests, and the slope stability was analyzed by using the finite element analysis model. The value coefficient of fiber soil slope was calculated based on the value coefficient method. The following conclusions were obtained by analyzing the value coefficient of the fiber soil embankment slope based on the response surface method:

(1) Compared with plain soil, the internal friction angle of fiber-reinforced soil showed no obvious change, but the cohesion was significantly enhanced. When analyzing the slope with ANSYS, the stability of the fiber-reinforced soil embankment slope showed a significant improvement.
(2) The results of the ANOVA indicated that the fiber diameter had the most significant effect on the value coefficient; the response surface illustrated the interaction between the three influencing factors, and the interaction between the fiber diameter and filling height is the most significant.
(3) The cost-effectiveness of the fiber soil slope was obtained by studying the strength reduction coefficient and value engineering method, and a regression polynomial with significant fitting effect was obtained to provide a reference for the actual project.

## Figures and Tables

**Table 1:** Basic physical properties of soil samples.

Properties	Specific Gravity	Maximum Dry Density (g/cm^3^)	Optimum Water Content (%)	Liquid Limit (%)	Plastic Limit (%)	Plasticity Index (%)
Value	2.42	1.725	12.2	34.3	25.0	9.5

**Table 2:** Shear strength parameters of fiber soil.

Fiber Diameter	0.02 mm	0.12 mm	0.2 mm	Plain Soil
Cohesion (Pa)	47,540	62,272	60,291	40,747
Internal Friction Angle (°)	21.186	21.653	22.568	22.053

**Table 3:** Code of influencing factors.

Coding Levels	Filling Height (h)/m	Fiber Diameter (d)/mm	Slope Angle (s)/°
−1	0	0.02	30
0	15	0.12	45
1	30	0.20	60

**Table 4:** Calculation table of value coefficients.

Run Number	Influencing Factors			Response Values
	A: h (m)	B: s (°)	C: d (mm)	Value Coefficient
1	0.00	60.00	0.02	7.7381
2	0.00	60.00	0.12	7.7381
3	0.00	30.00	0.12	7.19697
4	0.00	45.00	0.20	7.07071
5	15.00	60.00	0.02	6.92226
6	15.00	30.00	0.02	7.3776
7	15.00	45.00	0.12	7.86218
8	15.00	60.00	0.20	8.4317
9	15.00	30.00	0.20	7.8264
10	30.00	45.00	0.02	7.15488
11	30.00	30.00	0.12	7.24638
12	30.00	60.00	0.12	7.76398
13	30.00	45.00	0.20	7.80533

**Table 5:** ANOVA for response parameters.

Source	Sum of Squares	Degree of Freedom	Mean Square	F-Value	*p*-Value Prob > F
Model	2.38	10	0.24	18.37	0.0010
A-h	0.17	1	0.17	13.39	0.0106
B-s	9.92 × 10^−3^	1	9.92 × 10^−3^	0.77	0.4153
C-d	0.19	1	0.19	14.90	0.0084
AB	1.09 × 10^−4^	1	1.09 × 10^−4^	8.42 × 10^−3^	0.9299
AC	0.80	1	0.80	61.75	0.0002
BC	0.32	1	0.32	24.55	0.0026
A^2^	0.16	1	0.16	12.32	0.0127
B^2^	0.026	1	0.026	1.97	0.2099
C^2^	0.026	1	0.026	2.02	0.2055
A^2^C	0.55	1	0.55	42.42	0.0006
Residual	0.078	6	0.013	-	-
Cor Total	2.46	16	-	-	-

**Table 6:** ANOVA for models.

Statistic Factors	Value	Statistic Factors	Value
SD	0.11	R-Squared	0.9684
Mean	7.62	Adj R-Squared	0.9157
CV %	1.49	Pred R-Squared	−0.1629
PRESS	2.86	Adeq Precision	16.734

## Data Availability

The data presented in this study are available upon request from the corresponding author.
